# Exophytic Hepatic Hemangioma Mimicking a Right Adrenal Gland Mass: A Diagnostic Challenge

**DOI:** 10.7759/cureus.94498

**Published:** 2025-10-13

**Authors:** Arun Arumugam, Abdul Rahman Abualruz, Intisar Ghleilib, Ravishankar Pillenahalli Maheshwarappa

**Affiliations:** 1 Radiology, Augusta University Medical College of Georgia, Augusta, USA; 2 Radiology, Keck Medicine of USC, Los Angeles, USA; 3 Pathology and Laboratory Medicine, Augusta University Medical College of Georgia, Augusta, USA

**Keywords:** abdominal radiology, exophytic hepatic hemangioma, hemangioma, hepatic hemangioma, hepatic tumors

## Abstract

Hepatic hemangiomas are highly common benign tumors of the liver that are typically composed of vascular channels and are often discovered incidentally due to their asymptomatic nature. While the majority are intraparenchymal, exophytic hepatic hemangiomas are a rare occurrence that may mimic lesions from an adjacent organ. This case presents an unusual occurrence of an exophytic hepatic hemangioma in a 66-year-old male that closely mimicked a right adrenal gland mass on an incidental fluorine 18-fluorodeoxyglucose positron emission tomography/computed tomography or ¹⁸F-FDG PET/CT taken for the diagnosis of multiple myeloma. The atypical location and radiologic appearance complicated the initial diagnostic impression, highlighting the importance of considering exophytic hepatic hemangiomas in the differential diagnosis of adrenal and retroperitoneal masses. Recognition of this occurrence is critical to avoid unnecessary interventions and ensure appropriate patient management.

## Introduction

Hepatic hemangioma is the most common benign tumor of the liver, with a reported incidence reaching up to 7.3% in autopsy series and up to 20% in the general population [1,2]. These tumors are usually solitary, occur more frequently in women, and are most commonly located in the posterior segments of the right lobe [2]. The vast majority are small, asymptomatic, and incidentally detected on ultrasound, CT, or MRI performed for unrelated reasons [3]. While most hepatic hemangiomas are <3 cm in size, larger lesions (>10 cm), termed giant hemangiomas, may become symptomatic due to a mass effect or complications such as hemorrhage or rupture [1,4]. 

Exophytic hepatic hemangiomas, particularly those with a pedunculated morphology, are very rare, accounting for only a small fraction of all hemangioma cases. These atypical forms can be symptomatic or mimic neoplasms of adjacent organs on imaging, making diagnosis challenging. The difficulty arises from their unusual growth pattern, where the lesion extends beyond the liver surface with or without a detectable pedicle [5,6]. In such cases, differentiating hemangiomas from adrenal, renal, gastric, or retroperitoneal tumors is critical, as the treatment strategies vary considerably. 

Here, we describe an unusual case of an exophytic hepatic hemangioma arising from segment 7 of the liver and closely abutting the right adrenal gland. The abnormal location and radiologic appearance initially raised suspicion of an adrenal neoplasm, underscoring the importance of including exophytic hepatic hemangiomas in the differential diagnosis of suprarenal masses and highlighting the advantage of utilizing advanced imaging to guide treatment plans.

## Case presentation

**Figure 1:**
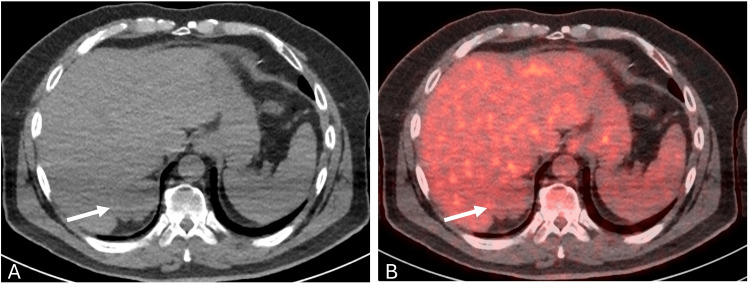
¹⁸F-FDG PET/CT images at the level of the right suprarenal lesion (A) Non-contrast CT image and (B) fused PET/CT image demonstrate a lobulated lesion noted posterosuperior to the right adrenal gland, closely abutting the adjacent segment 7 and right adrenal gland. The right adrenal gland shows low-grade FDG uptake (SUVmax of 3.1). ¹⁸F-FDG PET/CT, Fluorine 18-fluorodeoxyglucose positron emission tomography/computed tomography

A 66-year-old male with a past medical history of chronic kidney disease and hypertension was undergoing evaluation for possible multiple myeloma due to a one-year history of progressive numbness bilaterally in his feet. The patient underwent a whole-body fluorine 18-fluorodeoxyglucose positron emission tomography/computed tomography (¹⁸F-FDG PET/CT), which demonstrated a 5.4 × 3.6 cm lobulated mass in the right suprarenal region without suspicious FDG uptake. The lesion was noted to abut the adjacent liver segment 7 and the right adrenal gland and was incompletely characterized by non-contrast CT images (Figure 1).

**Figure 2:**
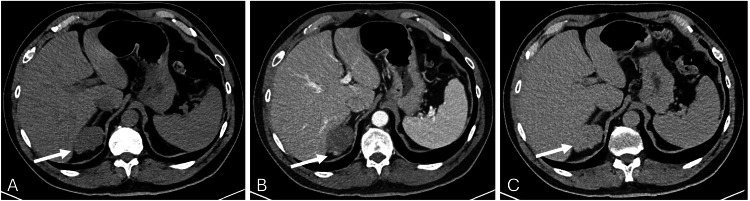
CT scan of the abdomen with and without contrast (adrenal protocol) (A) Non-contrast CT image demonstrates a lobulated hypodense lesion noted posterosuperior to the right adrenal gland, closely abutting the adjacent segment 7 and right adrenal gland (white arrow). (B) Post-contrast CT image in the porto-venous phase shows peripheral tiny nodular enhancement (white arrow). (C) A 15-minute delayed post-contrast image demonstrates homogeneous enhancement of the lesion (white arrow), consistent with centripetal contrast filling.

To further evaluate the lesion, a dedicated adrenal protocol CT was performed, which demonstrated peripheral nodular enhancement with centripetal filling on delayed phases, imaging features favoring a hemangioma. Although the lesion appeared to arise from the liver, its close relationship to the right adrenal gland made it difficult to definitively exclude an adrenal origin (Figure 2). 

**Figure 3:**
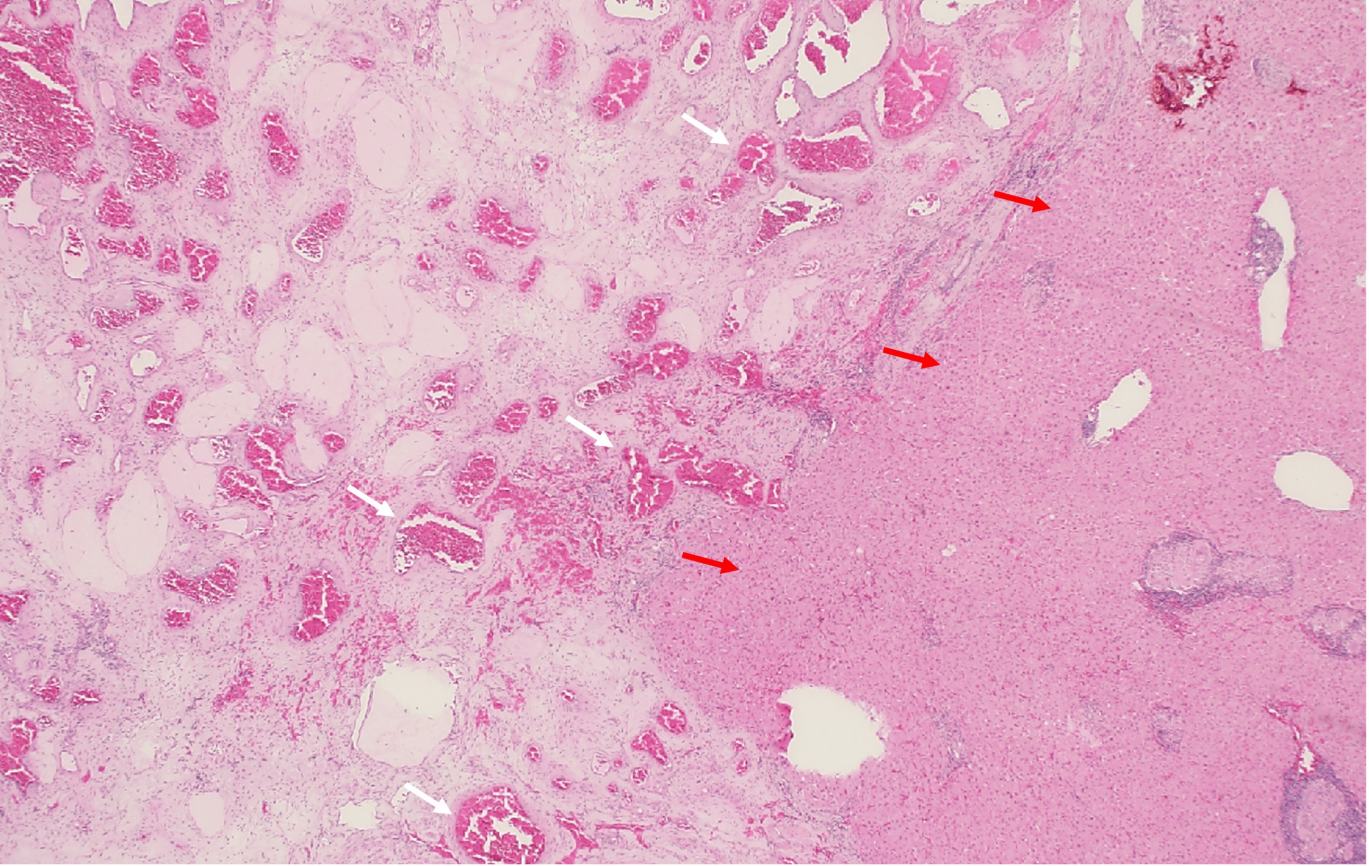
Histopathology image shows proliferation of variably sized, dilated, and thin-walled vessels (left of the image, indicated by white arrows) suggestive of hemangioma with adjacent liver tissue (right of the image, indicated by red arrows)

The adrenal laboratory panel, including metanephrines and normetanephrines, and a dexamethasone suppression test were within normal limits. After discussion of management options, the patient elected to proceed with surgical resection rather than surveillance. Pathologic examination confirmed the diagnosis of a hepatic hemangioma (Figure 3). 

The postoperative course was uneventful, and the patient was discharged on hospital day three. At follow-up, he denied any complications, and a CT scan at three months showed no evidence of residual or recurrent retroperitoneal mass.

## Discussion

Hepatic hemangiomas are typically characterized by their distinctive vascular profile on imaging. They typically show peripheral nodular enhancement with progressive centripetal fill-in on dynamic contrast CT or MRI, and high signal intensity on T2-weighted MR images [7,8]. These features usually allow for confident, non-invasive diagnosis. However, exophytic or pedunculated hemangiomas can deviate from classic imaging patterns, resulting in diagnostic uncertainty. 

In our case, the lesion was incompletely characterized by ^18^F-FDG PET/CT and closely abutted the right adrenal gland, raising concern for an adrenal neoplasm. The adrenal protocol CT demonstrated the classic enhancement pattern of a hemangioma; however, because of the close anatomic relationship to the adrenal gland, an adrenal origin could not be definitively excluded. This reflects a known limitation in cross-sectional imaging of exophytic masses, where thin pedicles may be radiologically occult [6]. Similar diagnostic pitfalls have been reported in the literature, with hemangiomas misdiagnosed as adrenal tumors, retroperitoneal neoplasms, or even gastric submucosal masses [5,9,10]. 

The clinical management of hepatic hemangiomas is usually conservative, as most are asymptomatic and carry a negligible risk of complications. However, large or symptomatic hemangiomas, particularly those with exophytic or pedunculated morphology, are often surgically resected due to the risks of torsion, infarction, or spontaneous rupture [11,12]. In our patient, despite the absence of hormonal activity and a benign enhancement pattern, the uncertainty in diagnosis and the proximity to the adrenal gland prompted surgical resection. Afterwards, pathology confirmed the diagnosis of hepatic hemangioma. 

This case emphasizes the importance of considering exophytic hepatic hemangiomas in the differential diagnosis of suprarenal masses, especially when imaging features suggest vascular lesions but the origin cannot be clearly defined. Awareness of this rare presentation can help prevent unnecessary interventions and guide appropriate treatment plans for patients.

## Conclusions

Although rare, exophytic hepatic hemangiomas can closely mimic adrenal or retroperitoneal lesions and present a significant diagnostic challenge. In our case, despite the use of advanced imaging techniques and adrenal functional workup, the lesion’s proximity to the right adrenal gland created ambiguity in determining its true origin. Surgical resection and subsequent histopathological confirmation ultimately established the diagnosis of a hepatic hemangioma. This case highlights the importance of considering exophytic hepatic hemangiomas in the differential diagnosis of suprarenal masses and underscores the role of correlating imaging findings with pathology for accurate diagnosis and appropriate management. 
